# Loneliness, living habits and hypertension among people over 60 years old in rural areas of Northeast China: Across-sectional study

**DOI:** 10.3389/fpubh.2025.1705167

**Published:** 2025-12-15

**Authors:** Shimeng Xiao, Zhimin Yi, Yi Wu, Ming Hao, Long Liu

**Affiliations:** 1 College of Design and Innovation, Tongji University, Shanghai, China; 2 School of Public Health and Health Management, Gannan Medical University, Ganzhou, China; 3 College of Art, Jinggangshan University, Ji'an, Jiangxi, China

**Keywords:** hypertension, loneliness, lifestyle, older population, China

## Abstract

**Background:**

High blood pressure is the leading cause of cardiovascular disease worldwide. The death rate from hypertension has almost doubled in China over the past decade as the country ages, with the rate increasing more sharply in rural areas than in urban areas. This study aimed to determine the influence of loneliness and lifestyle habits on blood pressure in older population individuals in Northeast China.

**Methods:**

In total, 1,085 older adults people aged over 60 years were recruited from rural areas of Northeast China for this study. The participants completed physical measurements (blood pressure, height, and weight), the Physical Activity Rating Scale 3 (PARS-3), the Loneliness Scale, and questionnaires that included information on the frequency of alcohol consumption and screen time.

**Results:**

The prevalence of high blood pressure and loneliness among women in Northeast China was higher than that among men. Women had lower levels of exercise than men. The results of multiple linear regression with hypertension as the dependent variable showed that screen time (*β* = 0.35, *p* < 0.01), Body Mass Index (BMI) (*β* = 0.31, *p* < 0.01), loneliness (*β* = 0.23, *p* < 0.01), and frequency of drinking (*β* = 0.12, *p* < 0.01) were risk factors for high blood pressure, while physical activity (*β* = −0.11, *p* < 0.01) was a protective factor against high blood pressure.

**Conclusion:**

Older adults people with more screen time had lower exercise scores, greater levels of loneliness, and were more likely to have higher blood pressure. The data presented here highlight the impact of loneliness and lifestyle habits on hypertension among older population in rural areas of Northeast China.

## 1 Introduction

According to 2018 data from the World Health Organization, approximately 26.4% of the global population has high blood pressure, with approximately 60% of these individuals residing in developing countries, including China (1). High blood pressure is a significant contributor to the global cardiovascular disease epidemic, leading to various complications such as stroke, acute coronary syndrome, chronic heart failure, and chronic kidney disease (2).

The prevalence and management of hypertension exhibit significant variation across countries and regions, with a decline in prevalence observed in wealthy countries and escalation noted in numerous low- and middle-income countries, where more than 1 billion individuals with hypertension reside (3, 4). Despite the increasing incidence and mortality rates of hypertension, the prevention and treatment of hypertension remain inadequate. A study conducted by the World Health Organization and Imperial College London in the United Kingdom revealed that less than one in five people with high blood pressure worldwide had their blood pressure adequately controlled (4). Hypertension not only affects the quality of life of patients but also imposes substantial mental and economic pressure on patients, their families, and society at large. Therefore, in today’s rapidly aging population, prioritizing the prevention and improvement of hypertension is crucial for improving the older population’s quality of life.

Psychosocial factors, particularly loneliness among older population individuals, substantially contribute to the onset of hypertension (5). Loneliness is defined as a subjective feeling of unhappiness or dissatisfaction caused by a lack of certain relationships (5, 6). Social disconnection and loneliness are associated with increased all-cause mortality, which is comparable to traditional risk factors like smoking (6, 7). Moreover, loneliness is a notable factor associated with high blood pressure. A study involving 1,880 oder adults Malaysians revealed that nearly one-third of participants reported experiencing high levels of loneliness, which significantly increased their likelihood of developing high blood pressure later in life (8).

Another study involving 1,362 Iranian seniors found that loneliness was prevalent in more than half of the participants and identified it as a risk factor for high blood pressure (6). However, research on loneliness and its correlation with high blood pressure has mainly been conducted on Western populations, thereby lacking unique insights into the Chinese context.

Physical activity may be one of the most promising non-drug methods for managing high blood pressure in oder adults individuals (9). Research indicates that exercise can delay the onset of high blood pressure (10, 11). Both aerobic and resistance exercises have demonstrated effectiveness in reducing blood pressure (10, 11). In contrast to drug treatments, which may entail side effects, high costs, and other drawbacks (5), brisk walking is a simple, inexpensive, and effective form of exercise, making it suitable for recommendation to society (10).

Studies have consistently demonstrated that screen time is associated with sedentary behavior (12). Sedentary behavior has been established as a substantial contributor to adverse physical and mental health outcomes in both children and adults (13, 14). Previously, prolonged screen time was primarily associated with children and young adults. However, with societal advancements, an increasing number of older population individuals are becoming proficient at using electronic devices. Smartphone-based medical monitoring, for instance, is employed for chronic disease management in middle-aged and older populations (12).

Diverse views exist regarding the relationship between alcohol consumption and high blood pressure. A study involving Japanese adults showed a positive association between alcohol consumption and high blood pressure, regardless of age or sex (15). However, other studies have shown that light and moderate drinking are not strongly associated with high blood pressure and are associated with improved cardiovascular outcomes compared to abstaining from alcohol (16). Further investigation is necessary to understand the reasons behind these disparities.

Over the past decade, China has witnessed a rapid increase in its older population and the number of oder adults individuals with hypertension. From 2007 to 2017, the death rate from hypertension in China nearly doubled, with a more pronounced increase observed in rural areas compared to urban areas (17). Oder adults residents of Northeast China experience prolonged winters, contributing to prevalent drinking habits among this demographic and reducing opportunities for outdoor activities. Furthermore, systolic blood pressure has been strongly associated with cardiovascular and cerebrovascular diseases (18).

A study has shown that men have higher systolic blood pressure levels before middle age, while women’s blood pressure increases significantly with age (19). Both men and women experience an increase in the aortic dilation index (a specific indicator for measuring central arterial blood pressure) that is directly related to age (19). Additionally, another study indicates that even without known cardiovascular diseases, aging and female gender are associated with an increase in vascular and myocardial stiffness. Among the possible determinants, the gender differences in hypertension can be regarded as the consequences of biological and behavioral issues (20).

Hence, this study aims to investigate the impact of loneliness and lifestyle habits on blood pressure among older population individuals in Northeast China.

## 2 Methods and materials

### 2.1 Study design

This study was conducted in accordance with the Helsinki Declaration. This study has been approved by the Ethics Committee (No. 2019129) during the investigation process. In accordance with these policies, investigators provided research participants with detailed explanations about the data being collected in this study and confirmed that it is being used only for scientific research purposes and not being disclosed to the public. All participants provided written electronic informed consent.

This study aimed to assess the impact of loneliness and lifestyle habits on blood pressure among older population individuals in Northeast China. The rural department of a district in Benxi city, located in Liaoning Province, Northeast China, was selected as the investigation area. The inclusion criteria were community residents aged 60 and above, who had complete health records at the community health service station, could clearly answer the questionnaire questions without obvious speech or hearing impairments, and voluntarily signed the informed consent form and cooperated with all the investigation procedures. The exclusion criteria included those who did not answer 20% or more of the total questions in the questionnaire, those with obvious logical confusion or incorrect answers, and those who had experienced major life events within 6 months before the investigation, such as the death of a close relative, a major disease diagnosis, severe damage to family property due to major natural disasters or accidents, etc. These situations may cause short-term fluctuations in psychological state and affect the stability of the research results. To recruit participants, we utilized a convenience sampling method. Specifically, we attracted oder adults individuals to participate in our survey by offering them free health check-ups. Every older population person who came for the health check-up was asked if they would be willing to take part in our survey. This method allowed us to reach a substantial number of oder adults participants within the community setting. Invitations were extended to older individuals visiting the regional health service for free medical examinations. Between April 2022 and October 2023, 1,200 seniors agreed to participate in the study. After excluding 115 incomplete or missing data, the complete data of the remaining 1,085 participants were included in the analysis. Sample-size determination was calculated with the use of G*Power 3.1.9.7 software, with five predictors, 95% power, and a significance level of 0.05, and a minimum of 1,073 participants were required to detect small effects (*f*^2^ = 0.02) in a multiple linear regression. So our sample size is sufficient for our multiple linear regression analysis.

### 2.2 Measurements

A practical electronic sphygmomanometer (J710, Omron; Japan) was utilized to uniformly measure blood pressure on the left wrist. Hypertension was diagnosed if the systolic blood pressure was ≥140 mmHg and/or diastolic blood pressure was ≥90 mmHg or if the patient had previously been diagnosed with hypertension and was taking blood pressure medications (21). The measurements were taken in a sitting position after at least 5 min of rest. Blood pressure was recorded three times consecutively with a 1-min interval between measurements. The mean of the second and third readings was used for analysis. Additionally, we have clarified that all blood pressure measurements were taken on the left arm. The data was collected during the examination period, and the final questionnaire results will be sent to the participants along with the physical examination results.

A height ruler with an accuracy of 0.1 cm (Seca 213, Seca Nihon; Germany) was utilized for height measurements. Height (0.1 kg precision) was measured using a body composition instrument (BC 601, Tanita; Japan). BMI was calculated based on height and weight.

### 2.3 Questionnaire content

#### 2.3.1 PARS-3

The Physical Activity Scale 3 (PARS-3) (22) was employed to evaluate the physical activity levels of rural oder adults individuals. The scale comprises three questions assessing the intensity, duration, and frequency of exercise. A 5-point scoring system was utilized to assess the intensity, frequency, and duration of exercise (1–5 points each). The total amount of exercise was calculated by multiplying the exercise intensity, duration, and frequency. Exercise was rated on a scale of 0–100 points, Categorized as follows: Light exercise: 0–19 points; Moderate exercise: 20–42 points; strenuous exercise: 43–100 points (23).

#### 2.3.2 Loneliness scale

Loneliness was measured using three questionnaires based on the revised UCLA Loneliness Scale, which has been validated for use in large epidemiological settings (24, 25). It comprised three items: “How often do you feel lonely?” “How often do you feel like you lack companionship?” Responses were categorized as 1 (almost never), 2 (sometimes), and 3 (often). The overall score was calculated, with a higher score indicating greater loneliness and a score exceeding 7 indicating a high level of loneliness (25).

### 2.4 Screen time

The screen time of the older population participants was obtained via questionnaires. Screen time, defined as the use of electronic products (such as mobile phone, computer, tablet, and TV). Self-designed questionnaires were used to collect participants’ self-reported screen time and other data. The self-reported screen time was the average duration (minutes per day) during the past week (26). And frequency of alcohol consumption was also assessed. Frequency of alcohol consumption was divided into three groups: (<1 time/month), (≥1 time/month and ≤2–4 times/month), and (≥2–3 times/week) (27, 28).

### 2.5 Statistical analysis

An independent samples *t*-test was utilized to investigate sex differences in the mean age, BMI, blood pressure, loneliness scores, exercise level scores, and screen time among participants. Chi-square tests were employed to compare the prevalence of high blood pressure and loneliness in men and women, as well as differences in the frequency and style of drinking. Tukey’s test was conducted to examine the differences in systolic blood pressure and average loneliness scores among participants with four different drinking styles. Correlation tests were employed to analyze the relationships among loneliness scores, exercise levels, and screen time in older population. For multiple regression analyses, systolic blood pressure served as the dependent variable, while BMI, total physical activity score, loneliness score, screen time, and frequency of alcohol consumption were predictors. In our study, we have a substantial number of potential predictors. Stepwise regression allows us to systematically evaluate these predictors and select the most significant ones, ensuring that our model is not overly complex and remains interpretable. Thus, variables were selected using the stepwise increase and decrease method, with a threshold *p* value of 0.20 calculated using the likelihood ratio test. All the parameter tests were performed on the premise that the data follow a normal distribution. Statistical significance was set at *p* < 0.05. JMP version 16.0 J (SAS Institute Inc., Cary, NC, USA) was used for all statistical analyses.

## 3 Results

### 3.1 Participants’ basic characteristics

The average age of the study participants was 71 years, with no significant sex differences observed in average age or BMI (Table 1). High blood pressure was prevalent in more than 60% of older population, with a higher prevalence noted among women compared to men (Table 1). Additionally, over 70% of oder adults individuals had a strong sense of loneliness, With women exhibiting higher levels of loneliness than men. Furthermore, less than 30% of older adults had exercise levels above the medium level, with women demonstrating lower levels of exercise compared to men (Table 1).

**Table 1:** Characteristics of the study subjects.

	Mean ± SD or *n* (%)		*p*
	Man (*n* = 509)	Woman (*n* = 576)	
Age	71.1 ± 4.6	71.1 ± 4.3	>0.05
BMI (kg/m^2^)	24.1 ± 3.2	24.0 ± 3.4	>0.05
Blood pressure			
Systolic pressure	143.0 ± 14.8	149.3 ± 21.2	<0.01
Diastolic pressure	78.8 ± 8.9	82.2 ± 10.4	<0.01
Hypertension			
Yes	313 (61.5%)	388 (67.4%)	<0.01
No	196 (38.5%)	188 (32.6%)	
Loneliness	6.3 ± 1.9	7.3 ± 1.6	<0.01
High loneliness level			
Yes	295 (58%)	374 (65%)	<0.01
No	214 (42%)	202 (35%)	
Physical activity score	18.9 ± 19.5	14.6 ± 18.0	<0.01
Low	335 (66%)	450 (78%)	<0.01
Medium	104 (20%)	64 (11%)	
High	70 (14%)	62 (11%)	
Screen time (min)	194.7 ± 85.3	230.0 ± 90.2	<0.01
Alcohol consumption (%)			
No drinking	209 (41%)	219 (38%)	>0.05
<1 time/month	229 (45%)	230 (40%)	
≥1 time/month and ≤2–4 times a month	61 (12%)	98 (17%)	
≥ 2–3 times a week	10 (2%)	29 (5%)	

### 3.2 Correlations between exercise scores, loneliness and screen time in older population

**Figure 1:**
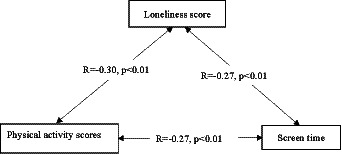
Correlations between exercise scores, loneliness and screen time in older adults.

**Figure 2:**
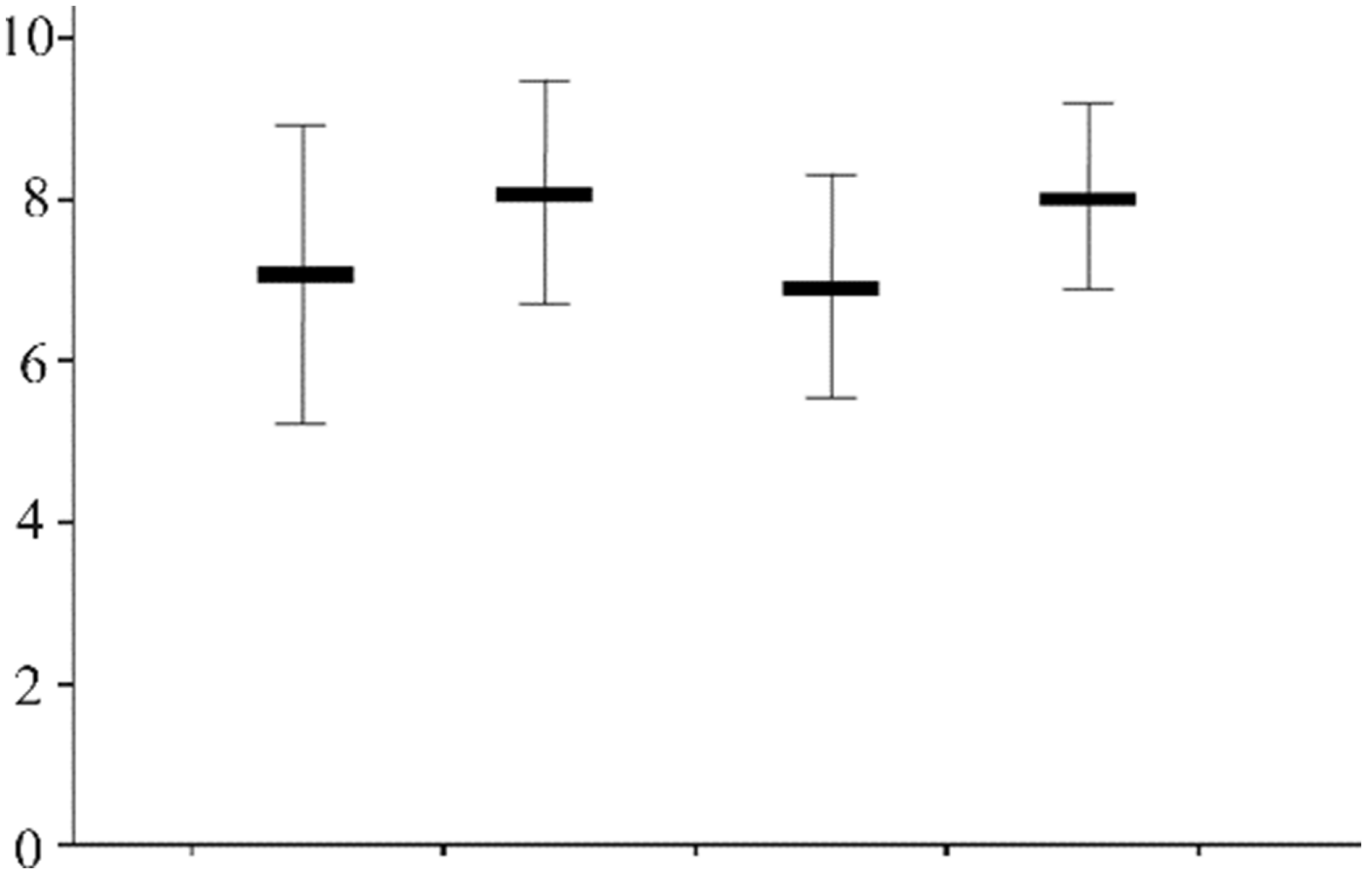
Drinking frequency and loneliness score (Mean, SD).

**Figure 3:**
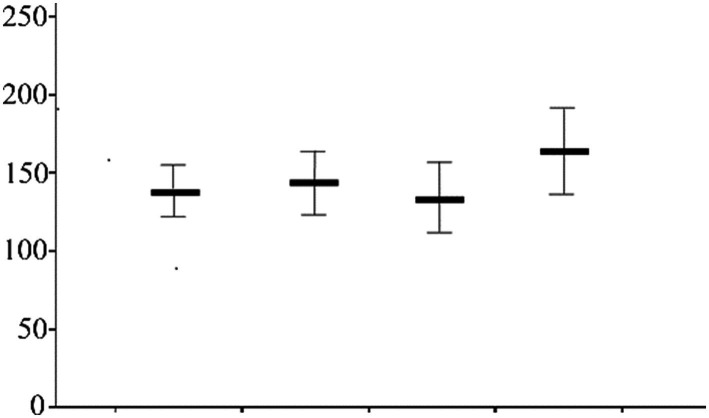
Drinking frequency and systolic pressure (Mean, SD).

**Figure 4:**
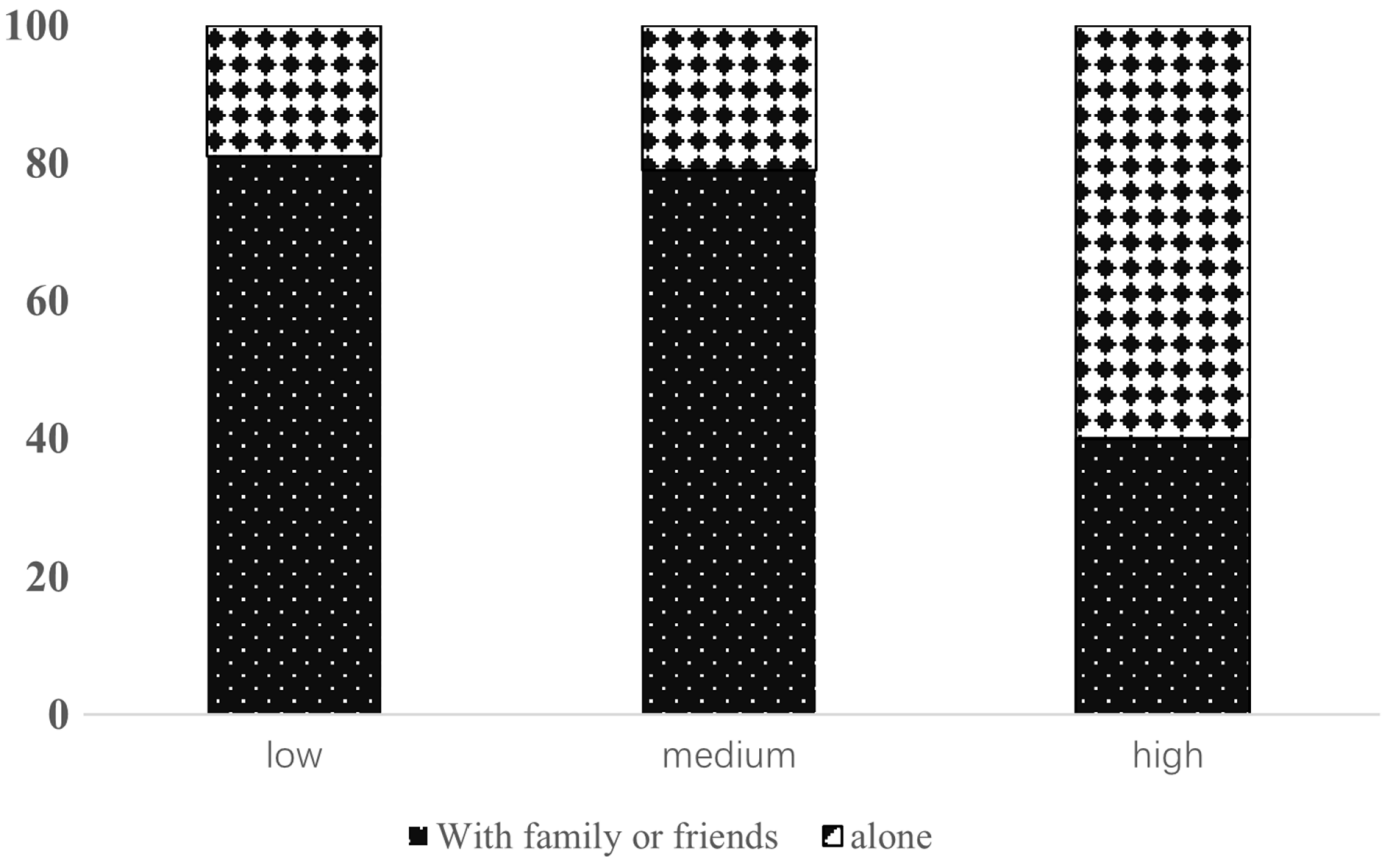
The drinking habits of people with different drinking frequencies.

A positive correlation was observed between the level of loneliness and exercise among oder adults individuals in Northeast China (Figure 1). Exercise time among older population decreased with increasing screen time (Figure 2). Concurrently, the loneliness level of older population individuals increased with increasing screen time (Figure 3). The average systolic blood pressure levels across different drinking frequency groups ranged from low to high, with the order being the medium-frequency, non-drinking, low-frequency, and high-frequency groups (Figure 2). Similarly, the average level of loneliness across different frequency groups ranged from low to high, with the order being medium-frequency, non-drinking, low-frequency, and high-frequency groups (Figure 3). The vast majority of oder adults drink alcohol with friends and family during gatherings (Figure 4).

The results of multiple linear regression analysis, with hypertension as the independent variable, revealed that screen time (*β* = 0.35, *p* < 0.01), BMI (*β* = 0.31, *p* < 0.01), loneliness (*β* = 0.23, *p* < 0.01), and alcohol consumption frequency (*β* = 0.12, *p* < 0.01) were identified as risk factors for hypertension (Table 2). Conversely, an exercise level of *β* = −0.11 (*p* < 0.01) was determined to be a protective factor against hypertension (Table 2).

**Table 2:** Factors that contributed to systolic pressure of older population (*n* = 1,085).

	*β*	*t*	VIF	*p*
Uncontrolled gender				
Screen time	0.35	15.72	1.46	<0.01
BMI	0.31	14.03	1.40	<0.01
Loneliness score	0.23	10.82	1.32	<0.01
Physical activity score	−0.11	−5.68	1.16	<0.01
Frequency of drinking	0.12	6.24	1.45	<0.01
Controlled gender				
Screen time	0.35	15.39	1.49	<0.01
BMI	0.32	14.03	1.40	<0.01
Loneliness score	0.23	10.27	1.39	<0.01
Physical activity score	−0.11	−5.59	1.17	<0.01
Frequency of drinking	0.12	6.24	1.03	<0.01

*R*^2^: 0.62; *p* < 0.01; RMES: 11.52.

## 4 Discussion

### 4.1 Gender differences

Previous studies have suggested that in middle-aged individuals, the incidence of hypertension is generally higher in men than in women (29, 30). Sex differences exist in the prevalence of hypertension among older population individuals. For example, one study found that the incidence of hypertension in men is 1.77 times higher than that in women (30).

Contrary with previous research, the current study findings demonstrated a greater incidence of hypertension in women compared to men (Table 1). In our study, the incidence of hypertension was high among both male and female older population individuals in the Northeast region of China (over 60%). This may be attributed to several factors. Firstly, the cold winter in the Northeast reduces outdoor activities and physical activity levels among the older population, which is negatively correlated with blood pressure. Additionally, the diet in the Northeast region is characterized by high salt and saturated fat intake, which is associated with the development of hypertension. Moreover, in terms of gender differences, the risk of hypertension is generally higher in men than in women at a younger age, but it reverses in older age, with women having a higher risk than men. This reversal typically occurs around the age of 60 (29). Noteworthy, there are inherent disparities in the incidence of high blood pressure between men and women. Studies have shown that for every 10 mmHg increase in systolic blood pressure, the incremental increase in cardiovascular diseases (e.g., coronary heart disease, ischemic heart disease, or myocardial infarction) is 15% in men and 25% in women (31). Sex differences are also evident in various conditions stemming from high blood pressure, such as heart failure, with women experiencing a greater burden of the disease (32). In summary, more attention should be paid to hypertension among older population women in Northeast China.

### 4.2 Loneliness level of older population individuals in Northeast China

The results of a pilot study showed that the prevalence of loneliness among older population individuals in rural China ranges from 25% to 78% (17). The results of this present study reveal that more than 60% of older adults in rural areas in Northeast China experience loneliness (Table 1), positioning it at a notably high level within the country. This percentage surpasses the reported 17% among adults in the United States and 30% among older Australians (33). The combination of China’s one-child policy and extensive rural–urban migration of working older population individuals between 1997 and 2015 has been identified as a significant contributor to the heightened loneliness among rural older populations (34). This phenomenon is particularly pronounced in Northeast China, where population decline is severe (35).

Previous studies have highlighted that older adults living with tend to experience lower levels of loneliness (36). However, in Northeast China, a substantial number of young people migrate to other provinces for work, making it difficult for older population individuals to interact with their children and grandchildren, thereby exacerbating loneliness levels among the older population in this region.

### 4.3 Relationship between loneliness and systolic blood pressure

The level of loneliness among women in Northeast China exceeded that among men (Table 1). Loneliness is considered an important factor in high blood pressure. A study involving 1,880 older population Malaysians identified loneliness as a major risk factor for high blood pressure (8). Similarly, a study of 797 adults in Fujian, China, underscored loneliness as a significant risk factor for high blood pressure (17). The stress-response model posits that psychological stress can lead to physiological responses that contribute to health outcomes, including hypertension. Loneliness, as a form of psychological stress, can activate the stress-response system, leading to increased blood pressure (37). This model suggests that chronic stress, such as that experienced by individuals who feel lonely, can result in sustained activation of the hypothalamic-pituitary-adrenal (HPA) axis, which in turn can lead to hypertension. Elevated cortisol levels, which may be seen in individuals experiencing loneliness, can contribute to hypertension through their effects on the renin-angiotensin system and adrenergic receptors (17). Our findings that loneliness is a significant risk factor for hypertension align with this model, indicating that the stress of social isolation may contribute to elevated blood pressure. The results of the multiple linear regression analysis indicated a positive correlation between the blood pressure of older population individuals and their loneliness score (Table 2), aligning with the findings of previous studies. The heightened level of loneliness among women may serve as a significant contributing factor to the higher incidence of hypertension in women compared to men.

### 4.4 Effect of exercise level on systolic blood pressure

The direct impact of exercise on hypertension among rural residents in Northeast China is shown in Table 2. Adequate exercise is recognized as a preventive and therapeutic measure for high blood pressure (17). However, there are unique considerations in rural areas. A study conducted among older population individuals in Fujian Province, located in South China, found no association between exercise levels and high blood pressure (17). Most older population people need to cultivate their own rice paddies, and intense physical labor reduces the effects of exercise on hypertension (17). In contrast to the southern region, where rice is cultivated thrice a year, the research area primarily focuses on corn cultivation, which has only one harvest period annually. Increased idle time may be an important reason for the correlation between exercise level and the incidence of hypertension in northern China. To effectively manage high blood pressure, some studies recommend engaging in at least 30 min of moderate-intensity aerobic exercise at least 3 days a week (10). However, less than 30% of older adults in the present study achieved moderate exercise levels. Insufficient exercise may be an important reason for the high incidence of hypertension among oder adults individuals in Northeast China. Additionally, women’s exercise scores were lower than those of men, which may be an important reason for the higher prevalence of hypertension in women.

### 4.5 Effect of loneliness on high blood pressure

Loneliness may reduce opportunities for physical activity. A study of 229 White, Black, and Hispanic men and women aged 50–68 years showed that high levels of loneliness may lead to lower physical activity rates (38). The results of this present study showed that older adults with higher loneliness scores had lower exercise levels, aligning with the findings of previous studies (Figure 1). Reducing loneliness may be pivotal in preventing high blood pressure in the older population. Beyond directly improving high blood pressure levels, reducing loneliness can also prevent the decline in physical activity, thereby averting the onset of high blood pressure.

### 4.6 Relationship between screen time and high blood pressure

Among adults from 10 European countries, watching TV for 1 h or more per day was associated with an increased risk of cardiovascular disease (39). Furthermore, this present study revealed that high screen time increased the risk of high blood pressure, supporting the findings of previous studies. A study of Chinese adults revealed that more than 40% of people aged over 45 engage in more than 2 h of screen time daily (40). The results of our study showed that all older adults had more than 2 h of screen time per day (Table 1). The average screen time was 213 min. Excessive screen time may contribute to a reduction in exercise duration and an increase in loneliness levels (Figure 1). Consequently, reducing screen time plays an important role in improving blood pressure among oder adults individuals in Northeast China.

Geotechnology has emerged as a multifaceted tool to tackle these challenges (41). While technologies such as wearable devices and health applications provide new avenues for monitoring and managing health, they also introduce complexities, especially concerning the social values and interactions of the older population (42). It is important to balance technology use with other enriching activities that foster community and interpersonal connections, ensuring a diversified approach to enhance the quality of life of older population individuals. This strategy acknowledges the diverse needs and values of the older population, who advocate a balanced lifestyle that reduces screen time while promoting more dynamic and socially engaging activities.

### 4.7 Relationship between alcohol consumption frequency and hypertension

The findings of this study revealed that moderate alcohol consumption improved hypertension (Figure 2). Previous studies have emphasized the role of moderate alcohol consumption in preventing and improving cardiovascular and cerebrovascular diseases (43). Alcohol consumption has been shown to be detrimental to cardiovascular health (10). The underlying reason for this paradox may be unrelated to drinking habits. Participants who moderately consumed alcohol reported experiencing less loneliness compared to those who abstained from alcohol (Figure 3). Regarding drinking patterns, the majority of older population individuals with moderate to low drinking frequencies consumed alcohol with relatives and friends (Figure 4). The social nature of drinking may contribute to lower blood pressure by reducing loneliness among older adults.

### 4.8 Limitations

This study had several limitations. First, it had a small sample size and included only older adults individuals aged over 60 years. Additionally, as a cross-sectional study, causal inferences could not be established. Longitudinal studies are recommended to better understand the causal relationships between risk factors and hypertension. This approach would provide more robust evidence for public health interventions. Because the subjects we selected were drawn from the community, there is some sampling error. Future studies should therefore consider more diverse recruitment strategies, such as community-based random sampling, to minimize selection bias. Moreover, this study investigated only rural areas in Northeast China. Therefore, future studies should focus on southern China and its urban areas. Moreover, to address the potential limitations of the three-item scale, future research should consider using more comprehensive measures of loneliness that can better capture its multidimensional nature. For instance, the original 20-item UCLA Loneliness Scale or the Multidimensional Loneliness Scale (MLS) could be employed to provide a more nuanced understanding of loneliness. These scales include additional dimensions such as family estrangement, loss of attachment figures, and intrapersonal emptiness, which might be particularly relevant in collectivist cultures. Moreover, despite the importance of cultural dimensions, our study did not specifically analyze the impact of these cultural factors on loneliness. Future research should incorporate more nuanced cultural analyses to better understand the multifaceted nature of loneliness in rural China. This could include qualitative studies to explore the lived experiences of loneliness and how cultural values and practices influence these experiences. Additionally, longitudinal studies that track changes in loneliness over time in the context of ongoing social and economic changes could provide deeper insights into the dynamics of loneliness in rural areas.

## 5 Conclusion

The findings of this study indicate that women in Northeast China exhibit higher rates of hypertension and loneliness compared to men. Furthermore, women demonstrate lower levels of exercise than men. These results underscore the impact of loneliness levels and lifestyle habits on hypertension among the older adults population residing in rural areas of Northeast China.

## Data Availability

The original contributions presented in the study are included in the article/supplementary material, further inquiries can be directed to the corresponding authors.

## References

[1] Herawati I Mat Ludin AF M M Ishak I Farah NMF. Breathing exercise for hypertensive patients: a scoping review. Front Physiol. (2023) 14:1048338. doi: 10.3389/fphys.2023.1048338, 36760529 PMC9905130

[2] Kalibala J Pechère-Bertschi A Desmeules J. Gender differences in cardiovascular pharmacotherapy-the example of hypertension: a Mini review. Front Pharmacol. (2020) 11:564. doi: 10.3389/fphar.2020.00564, 32435193 PMC7218117

[3] Mills KT Stefanescu A He J. The global epidemiology of hypertension. Nat Rev Nephrol. (2020) 16:223–37. doi: 10.1038/s41581-019-0244-2, 32024986 PMC7998524

[4] NCD Risk Factor Collaboration (NCD-RisC). Worldwide trends in hypertension prevalence and progress in treatment and control from 1990 to 2019: a pooled analysis of 1201 population-representative studies with 104 million participants. Lancet. (2021) 398:957–80. doi: 10.1016/s0140-6736(21)01330-1, 34450083 PMC8446938

[5] Abolfathi M Hajimoradloo A Ghorbani R Zamani A. Effect of starvation and refeeding on digestive enzyme activities in juvenile roach, *Rutilus rutilus* caspicus. Comp Biochem Physiol A Mol Integr Physiol. (2012) 161:166–73. doi: 10.1016/j.cbpa.2011.10.020, 22062799

[6] Yousefi Z Id S Sameie S Taheri S Araj-Khodaei M Sanaie S . Loneliness as a predictor of hypertension in older adults: the TOPS study. Int J Aging. (2023) 1:7. doi: 10.34172/ija.2023.e7

[7] Holt-Lunstad J Robles TF Sbarra DA. Advancing social connection as a public health priority in the United States. Am Psychol. (2017) 72:517–30. doi: 10.1037/amp0000103, 28880099 PMC5598785

[8] Momtaz YA Hamid TA Yusoff S Ibrahim R Chai ST Yahaya N . Loneliness as a risk factor for hypertension in later life. J Aging Health. (2012) 24:696–710. doi: 10.1177/0898264311431305, 22422758

[9] Souza LR Vicente JB Melo GR Moraes VC Olher RR Sousa IC . Acute hypotension after moderate-intensity handgrip exercise in hypertensive elderly people. J Strength Cond Res. (2018) 32:2971–7. doi: 10.1519/jsc.0000000000002460, 29384998

[10] Biddinger KJ Emdin CA Haas ME Wang M Hindy G Ellinor PT . Association of Habitual Alcohol Intake with Risk of cardiovascular disease. JAMA Netw Open. (2022) 5:e223849. doi: 10.1001/jamanetworkopen.2022.3849, 35333364 PMC8956974

[11] Saco-Ledo G Valenzuela PL Ruiz-Hurtado G Ruilope LM Lucia A. Exercise reduces ambulatory blood pressure in patients with hypertension: a systematic review and Meta-analysis of randomized controlled trials. J Am Heart Assoc. (2020) 9:e018487. doi: 10.1161/jaha.120.018487, 33280503 PMC7955398

[12] Zhang Y Wang J Lu X Che B Yu J. The associated factors of prolonged screen time and using electronic devices before sleep among elderly people in Shaanxi Province of China: a cross-sectional study. Int J Environ Res Public Health. (2021) 18:7020. doi: 10.3390/ijerph18137020, 34209159 PMC8297076

[13] Biddle SJ Gorely T Stensel DJ. Health-enhancing physical activity and sedentary behaviour in children and adolescents. J Sports Sci. (2004) 22:679–701. doi: 10.1080/02640410410001712412, 15370482

[14] Hamilton MT Hamilton DG Zderic TW. Role of low energy expenditure and sitting in obesity, metabolic syndrome, type 2 diabetes, and cardiovascular disease. Diabetes. (2007) 56:2655–67. doi: 10.2337/db07-0882, 17827399

[15] Ikehara S Iso H. Alcohol consumption and risks of hypertension and cardiovascular disease in Japanese men and women. Hypertens Res. (2020) 43:477–81. doi: 10.1038/s41440-020-0417-1, 32203447

[16] Mayl JJ German CA Bertoni AG Upadhya B Bhave PD Yeboah J . Association of Alcohol Intake with Hypertension in type 2 diabetes mellitus: the ACCORD trial. J Am Heart Assoc. (2020) 9:e017334. doi: 10.1161/jaha.120.017334, 32900264 PMC7726983

[17] Yazawa A Inoue Y Yamamoto T Watanabe C Tu R Kawachi I. Can social support buffer the association between loneliness and hypertension? A cross-sectional study in rural China. PLoS One. (2022) 17:e0264086. doi: 10.1371/journal.pone.0264086, 35180267 PMC8856532

[18] Rabkin SW Mathewson AL Tate RB. Predicting risk of ischemic heart disease and cerebrovascular disease from systolic and diastolic blood pressures. Ann Intern Med. (1978) 88:342–5. doi: 10.7326/0003-4819-88-3-342, 629496

[19] Hayward CS Kelly RP. Gender-related differences in the central arterial pressure waveform. J Am Coll Cardiol. (1997) 30:1863–71. doi: 10.1016/s0735-1097(97)00378-1, 9385920

[20] Redfield MM Jacobsen SJ Borlaug BA Rodeheffer RJ Kass DA. Age- and gender-related ventricular-vascular stiffening: a community-based study. Circulation. (2005) 112:2254–62. doi: 10.1161/circulationaha.105.541078, 16203909

[21] Castilla-Guerra L Fernández-Moreno MC. Update on the management of hypertension for secondary stroke prevention. Eur Neurol. (2012) 68:1–7. doi: 10.1159/000336836 22627064

[22] Yang G Li Y Liu S Liu C Jia C Wang S. Physical activity influences the mobile phone addiction among Chinese undergraduates: the moderating effect of exercise type. J Behav Addict. (2021) 10:799–810. doi: 10.1556/2006.2021.00059, 34546969 PMC8997213

[23] Gu S Zhang X Peng Y. A serial mediation model of physical exercise and loneliness: the role of perceived social support and resilience. BMC Geriatr. (2024) 24:811. doi: 10.1186/s12877-024-05407-1, 39369186 PMC11452978

[24] Russell D Peplau LA Cutrona CE. The revised UCLA loneliness scale: concurrent and discriminant validity evidence. J Pers Soc Psychol. (1980) 39:472–80. doi: 10.1037//0022-3514.39.3.472, 7431205

[25] Boehlen FH Maatouk I Friederich HC Schoettker B Brenner H Wild B. Loneliness as a gender-specific predictor of physical and mental health-related quality of life in older adults. Qual Life Res. (2022) 31:2023–33. doi: 10.1007/s11136-021-03055-1, 34859354 PMC9188519

[26] Ye H Wang Y Xu S Tu J Hao M Zhou X. The effects of body dissatisfaction, lifestyle, and loneliness on emotional eating among older adults in Northeast China. Aging Ment Health. (2025) 29:1515–24. doi: 10.1080/13607863.2025.2479188, 40135682

[27] Zhao J Wang X Xu S Yan W Wang J Wang E . The influence of lifestyle habits on levels of depression among rural middle school students in northeastern China. Front Public Health. (2024) 12:1293445. doi: 10.3389/fpubh.2024.1293445, 38347930 PMC10859412

[28] Yoo JI Ha YC Lee YK Hana C Yoo MJ Koo KH. High prevalence of sarcopenia among binge drinking elderly women: a nationwide population-based study. BMC Geriatr. (2017) 17:114. doi: 10.1186/s12877-017-0507-3 28558678 PMC5450303

[29] Yeo WJ Abraham R Surapaneni AL Schlosser P Ballew SH Ozkan B . Sex differences in hypertension and its management throughout life. Hypertension. (2024) 81:2263–74. doi: 10.1161/hypertensionaha.124.22980, 39229711 PMC11483212

[30] Defianna SR Santosa A Probandari A Dewi FST. Gender differences in prevalence and risk factors for hypertension among adult populations: a cross-sectional study in Indonesia. Int J Environ Res Public Health. (2021) 18:6259. doi: 10.3390/ijerph18126259, 34207848 PMC8296037

[31] Wei YC George NI Chang CW Hicks KA. Assessing sex differences in the risk of cardiovascular disease and mortality per increment in systolic blood pressure: a systematic review and Meta-analysis of follow-up studies in the United States. PLoS One. (2017) 12:e0170218. doi: 10.1371/journal.pone.0170218, 28122035 PMC5266379

[32] Connelly PJ Currie G Delles C. Sex differences in the prevalence, outcomes and Management of Hypertension. Curr Hypertens Rep. (2022) 24:185–92. doi: 10.1007/s11906-022-01183-8, 35254589 PMC9239955

[33] Petitte T Mallow J Barnes E Petrone A Barr T Theeke L. A systematic review of loneliness and common chronic physical conditions in adults. Open Psychol J. (2015) 8:113–32. doi: 10.2174/1874350101508010113, 26550060 PMC4636039

[34] Luo Y Waite LJ. Loneliness and mortality among older adults in China. J Gerontol B Psychol Sci Soc Sci. (2014) 69:633–45. doi: 10.1093/geronb/gbu007, 24550354 PMC4049147

[35] You H Yang J Xue B Xiao X Xia J Jin C . Spatial evolution of population change in Northeast China during 1992–2018. Sci Total Environ. (2021) 776:146023. doi: 10.1016/j.scitotenv.2021.146023

[36] Chokkanathan S. Prevalence of and risk factors for loneliness in rural older adults. Australas J Ageing. (2020) 39:e545–51. doi: 10.1111/ajag.12835, 33098243

[37] Hutto L Odlum M Theeke L. A systematic review of quantitative studies of depression and loneliness in black women with hypertension in the United States. J Racial Ethn Health Disparities. (2025). doi: 10.1007/s40615-025-02541-3, 40660030

[38] Hawkley LC Thisted RA Cacioppo JT. Loneliness predicts reduced physical activity: cross-sectional & longitudinal analyses. Health Psychol. (2009) 28:354–63. doi: 10.1037/a0014400, 19450042 PMC2791498

[39] Shiue I. Duration of daily TV/screen watching with cardiovascular, respiratory, mental and psychiatric health: Scottish health survey, 2012-2013. Int J Cardiol. (2015) 186:241–6. doi: 10.1016/j.ijcard.2015.03.259, 25828126

[40] Su Y Li X Li H Xu J Xiang M. Association between sedentary behavior during leisure time and excessive weight in Chinese children, adolescents, and adults. Nutrients. (2023) 15:424. doi: 10.3390/nu15020424, 36678295 PMC9867297

[41] Smith CD Mennis J. Incorporating geographic information science and technology in response to the COVID-19 pandemic. Prev Chronic Dis. (2020) 17:E58. doi: 10.5888/pcd17.200246, 32644920 PMC7367069

[42] Luo M Ding D Bauman A Negin J Phongsavan P. Social engagement pattern, health behaviors and subjective well-being of older adults: an international perspective using WHO-SAGE survey data. BMC Public Health. (2020) 20:99. doi: 10.1186/s12889-019-7841-7, 31973695 PMC6979381

[43] Rimm EB Klatsky A Grobbee D Stampfer MJ. Review of moderate alcohol consumption and reduced risk of coronary heart disease: is the effect due to beer, wine, or spirits. BMJ. (1996) 312:731–6. doi: 10.1136/bmj.312.7033.731, 8605457 PMC2350477

